# Validation of a Web-Based Planning Tool for Percutaneous Cryoablation of Renal Tumors

**DOI:** 10.1007/s00270-020-02634-y

**Published:** 2020-09-15

**Authors:** Tim J. van Oostenbrugge, Jan Heidkamp, Michael Moche, Phil Weir, Panchatcharam Mariappan, Ronan Flanigan, Mika Pollari, Stephen Payne, Marina Kolesnik, Sjoerd F. M. Jenniskens, Jurgen J. Fütterer

**Affiliations:** 1 Department of Urology, Radboud University Medical Center, P.O. box 9101, 6500 HB Nijmegen, The Netherlands; 2 Department of Radiology and Nuclear Medicine, Radboud University Medical Center, Nijmegen, The Netherlands; 3 Department of Diagnostic and Interventional Radiology, University Hospital, Leipzig, Germany; 4 NUMA Engineering Services Ltd, Dublin, Ireland; 5 Department of Neuroscience and Biomedical Engineering, Aalto University, Espoo, Finland; 6 Department of Engineering Science, Institute of Biomedical Engineering, University of Oxford, Oxford, UK; 7 Fraunhofer - FIT - Institute for Applied Information Technology, Sankt-Augustin, Germany; 8 MIRA, Institute for Biomedical Technology and Technical Medicine, University of Twente, Enschede, The Netherlands; 9 Department of Mathematics, Indian Institute of Technology, Tirupati, India

**Keywords:** Cryosurgery, Kidney neoplasms, Computer-assisted image processing, Intraoperative monitoring, Preoperative care

## Abstract

**Purpose:**

To validate a simulation environment for virtual planning of percutaneous cryoablation of renal tumors.

**Materials and Methods:**

Prospectively collected data from 19 MR-guided procedures were used for validation of the simulation model. Volumetric overlap of the simulated ablation zone volume (Σ) and the segmented ablation zone volume (*S*; assessed on 1-month follow-up scan) was quantified. Validation metrics were DICE Similarity Coefficient (DSC; the ratio between twice the overlapping volume of both ablation zones divided by the sum of both ablation zone volumes), target overlap (the ratio between the overlapping volume of both ablation zones to the volume of *S*; low ratio means *S* is underestimated), and positive predictive value (the ratio between the overlapping volume of both ablation zones to the volume of Σ; low ratio means *S* is overestimated). Values were between 0 (no alignment) and 1 (perfect alignment), a value > 0.7 is considered good.

**Results:**

Mean volumes of *S* and Σ were 14.8 cm^3^ (± 9.9) and 26.7 cm^3^ (± 15.0), respectively. Mean DSC value was 0.63 (± 0.2), and ≥ 0.7 in 9 cases (47%). Mean target overlap and positive predictive value were 0.88 (± 0.11) and 0.53 (± 0.24), respectively. In 17 cases (89%), target overlap was ≥ 0.7; positive predictive value was ≥ 0.7 in 4 cases (21%) and < 0.6 in 13 cases (68%). This indicates *S* is overestimated in the majority of cases.

**Conclusion:**

The validation results showed a tendency of the simulation model to overestimate the ablation effect. Model adjustments are necessary to make it suitable for clinical use.

## Introduction

Thermal ablative therapies for small renal masses (SRMs; < 4 cm) are an alternative treatment for nephron-sparing surgery (NSS) [1]. With fewer complications reported compared to the laparoscopic approach, the image-guided percutaneous approach for this treatment is established [2]. Despite the availability of larger studies with recurrence-free survival exceeding 85% after longer follow-up, long-term oncological outcomes for percutaneous cryoablation are under debate [3–5]. Two recent meta-analyses suggest higher local recurrence rates for cryoablation compared to partial nephrectomy; whereas, more recent studies show that cryoablation challenges partial nephrectomy for local control of cT1a renal tumors [6–8].

Currently, there is limited software available for interventional radiologists to plan the procedure and to predict procedural outcomes [9]. Obtaining full tumor coverage with enough margin is imperative and depends on the type, configuration, and the number of needles used. The physician makes these decisions based on the predicted ice ball formation from a single needle provided by the manufacturer and experience with the equipment used [10, 11]. Without proper support of dedicated software, physiological components such as organ tissue characteristics and blood flow are difficult to take into account [12, 13]. Once the ablation has started and the ice ball is formed, the possibility of needle replacement is obliterated. This emphasizes the importance of pre-procedural planning to ensure radical treatment.

In April 2013, the Go-SMART project started with the aim to build a generic open-source software simulation environment to be used for planning of image-guided percutaneous cancer treatment modalities [14]. The workflows of the environment are designed to enable minimal invasive procedure planning in advance by the interventionalist using a pre-interventional diagnostic scan only. A part of the project was to develop and incorporate a workflow for planning percutaneous cryoablation of renal tumors in the web environment. This included image segmentation and registration tools as well as a treatment simulation model with the ability for validation of this model.

The aim of our study was to validate the simulation model for virtual planning of percutaneous cryoablation of renal tumors. The model was designed based on two variables: an equitation modelling temperature development during ablation and a prediction of tissue response based on physiological properties.

## Materials and Methods

**Fig. 1:**
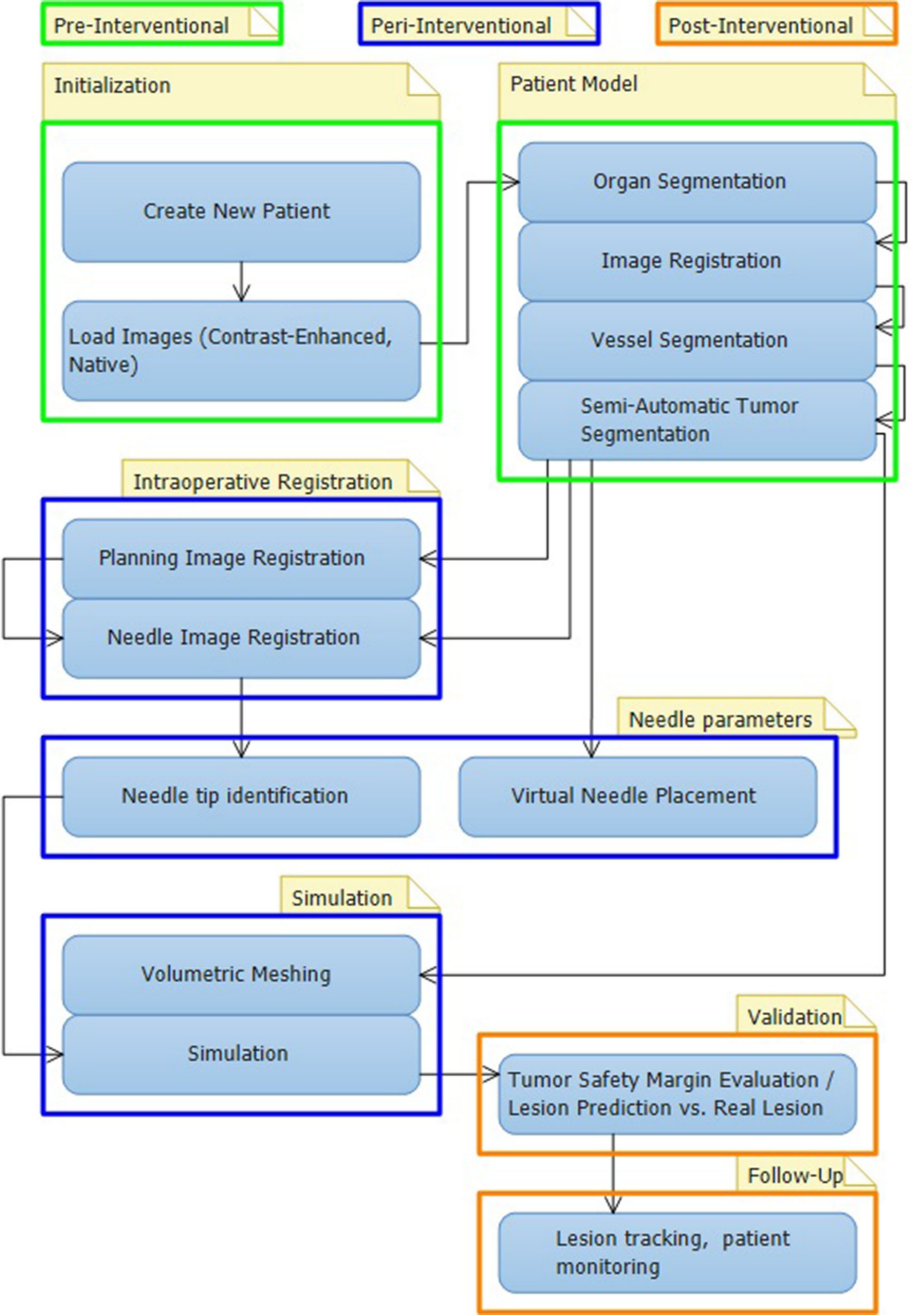
Workflow of web-based environment

This study was IRB approved. The development of the web-based environment (freely available through (https://smart-mict.eu/)) is extensively described [14]. In short, a simulation tool using a multi-scale physiological model was developed to predict the result of the treatments in terms of ablation zone size and shape. To calculate the first-order effect of the cryoablation, a modified Pennes bioheat equation with added perfusion term was used. This model is based on the density, specific heat capacity, heat conductivity, and temperature of the perfused tissue (renal tumor), heat flux due to the ablation instrument, and the norming effect of tissue perfusion. The norming effect on itself was based on the local fraction of cells considered dead, the perfusion coefficient, material property of renal tumor tissue, standard body temperature, current local temperature, and the density and specific heat capacity of blood [15]. Within the modified Pennes bioheat equation, physical properties change (liquid to solid) due to the expanding ice ball was taken into account. This resulted in the use of adjusted heat capacity and thermal conductivity [14]. For the cell death model under hypothermia, a simple empirical isotherm was used. Extensive discussion of the theory behind the mathematical model used for simulating is published elsewhere [13]. Validation tools were incorporated to verify the predicted treatment result based on true post-operative control images of treated patients. The web-based environment workflow is outlined in Fig. 1 and comprehensively described below.

### Workflow for Pre-procedural Simulation

**Fig. 2:**
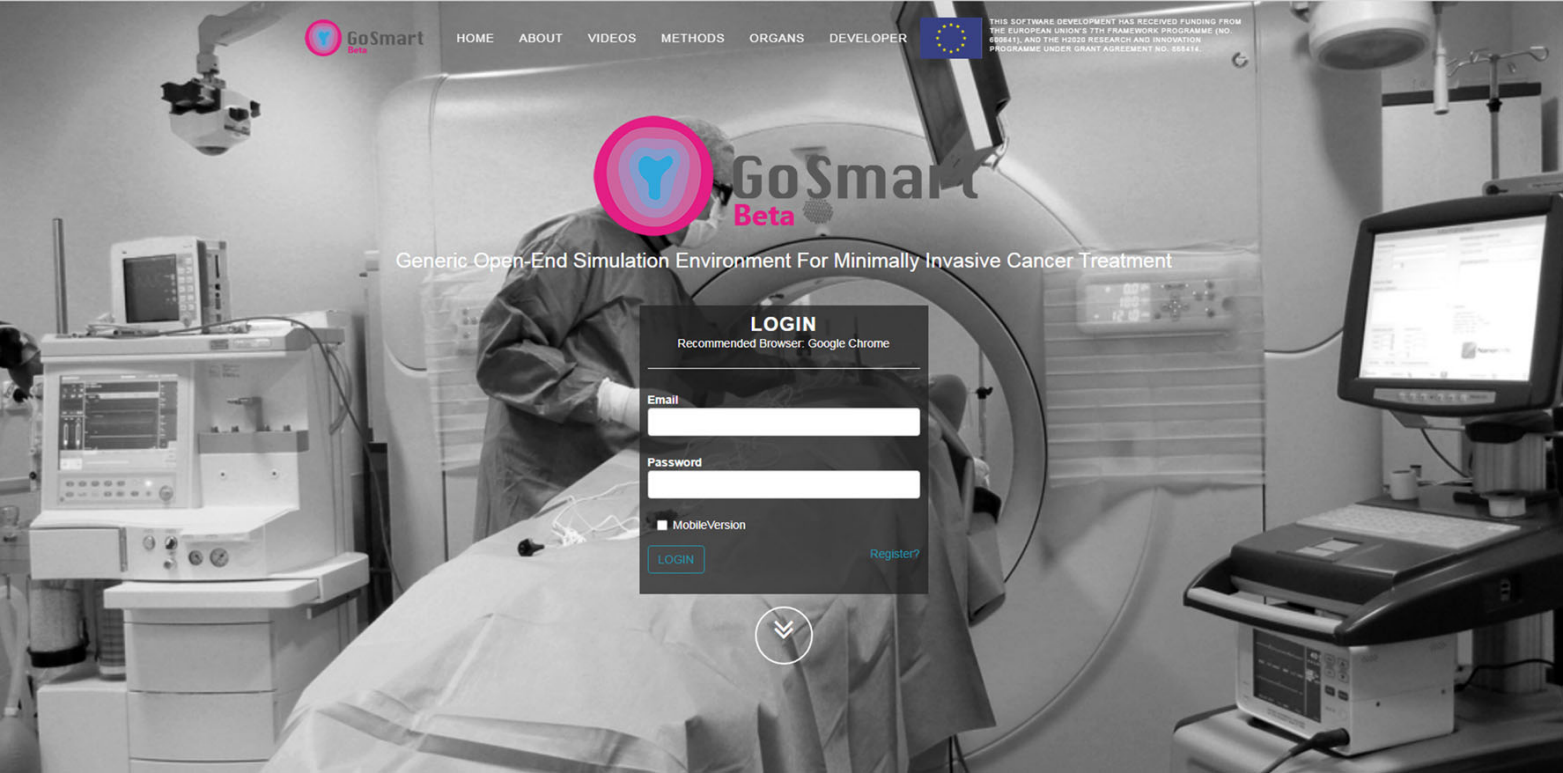
Startpage of web environment

**Fig. 3:**
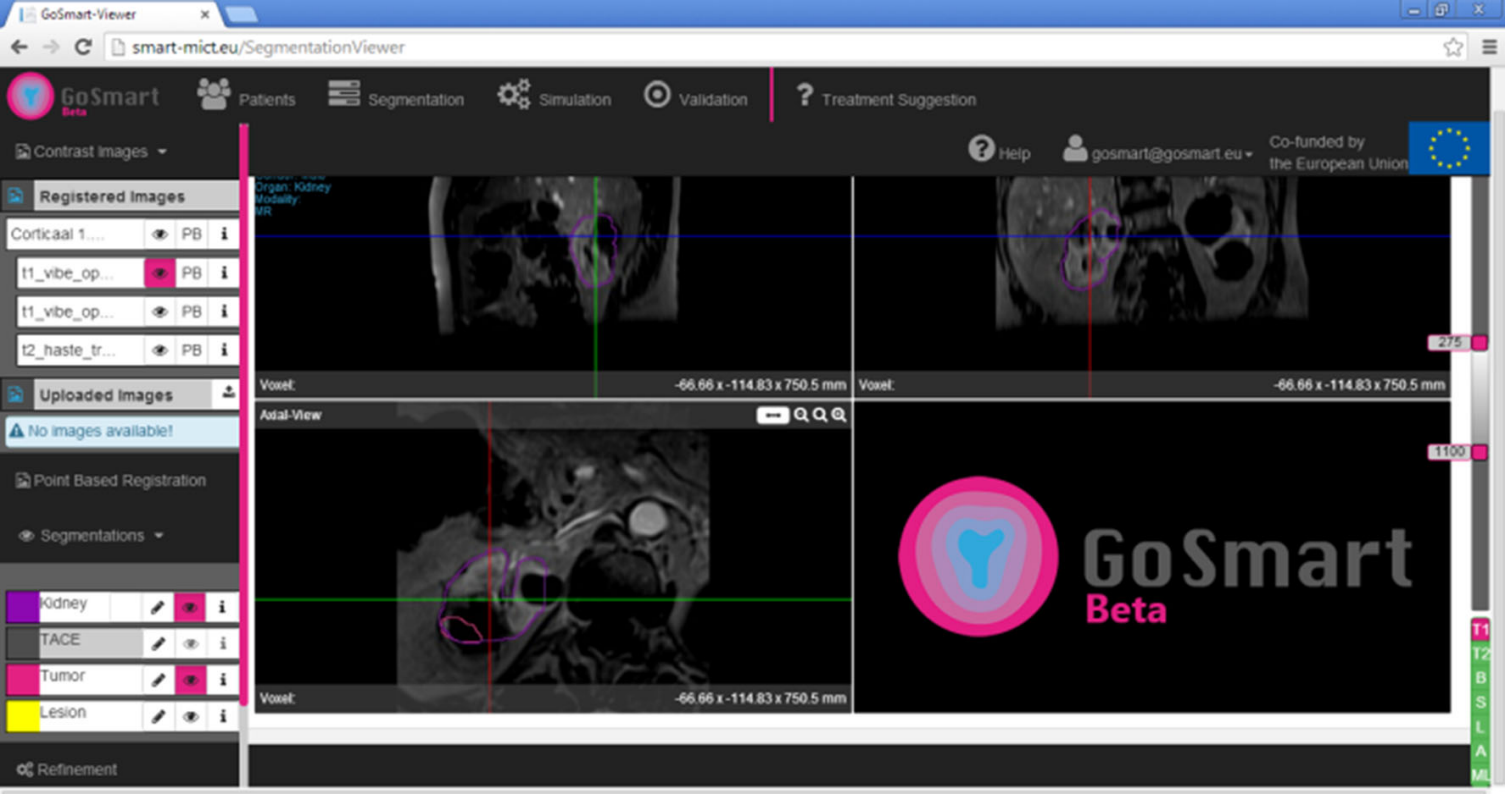
Web environment viewer showing MRI in three orthogonal planes. Available image series and tools are shown in bars on the left

**Fig. 4:**
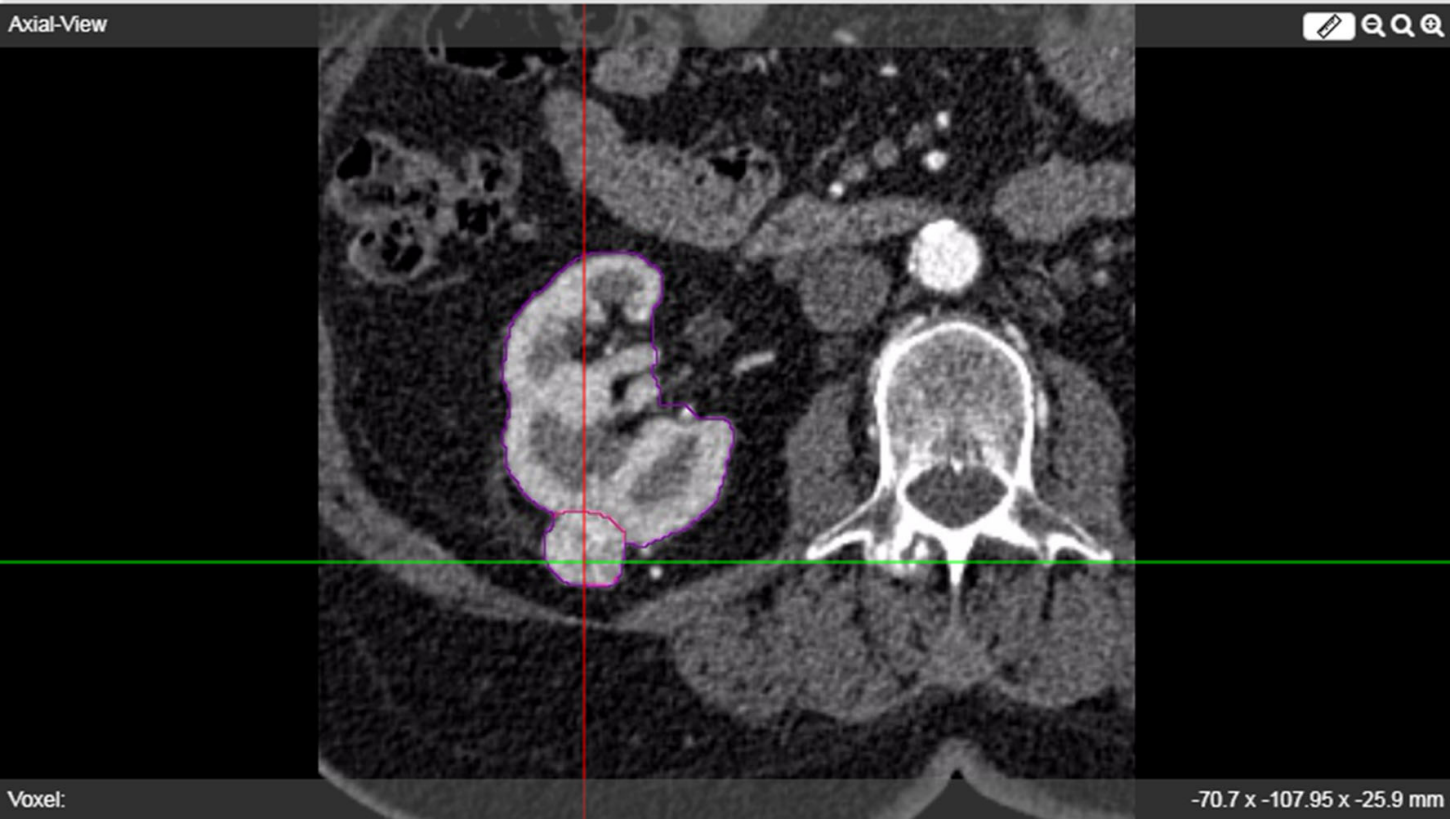
Contrast enhanced, pre-operative axial CT image in corticomedullary phase showing automated segmentation results of kidney (purple) and tumor (pink) in the viewer

As required for treatment planning, anonymous diagnostic contrast-enhanced cross-sectional CT and MRI scans are uploaded to a personal user account in separate patient folders. Used pre-interventional imaging was susceptible to slight parameter differences because treated patients were often referred from elsewhere and renewed diagnostic imaging was not always considered necessary. The kidney and the tumor are automatically segmented on the preoperative images using a seed point approach (Figs. 2, 3, 4). The automated segmentation can be adjusted manually by the user. Next, up to 9 virtual needles can be placed and a simulation can be started.

**Fig. 5:**
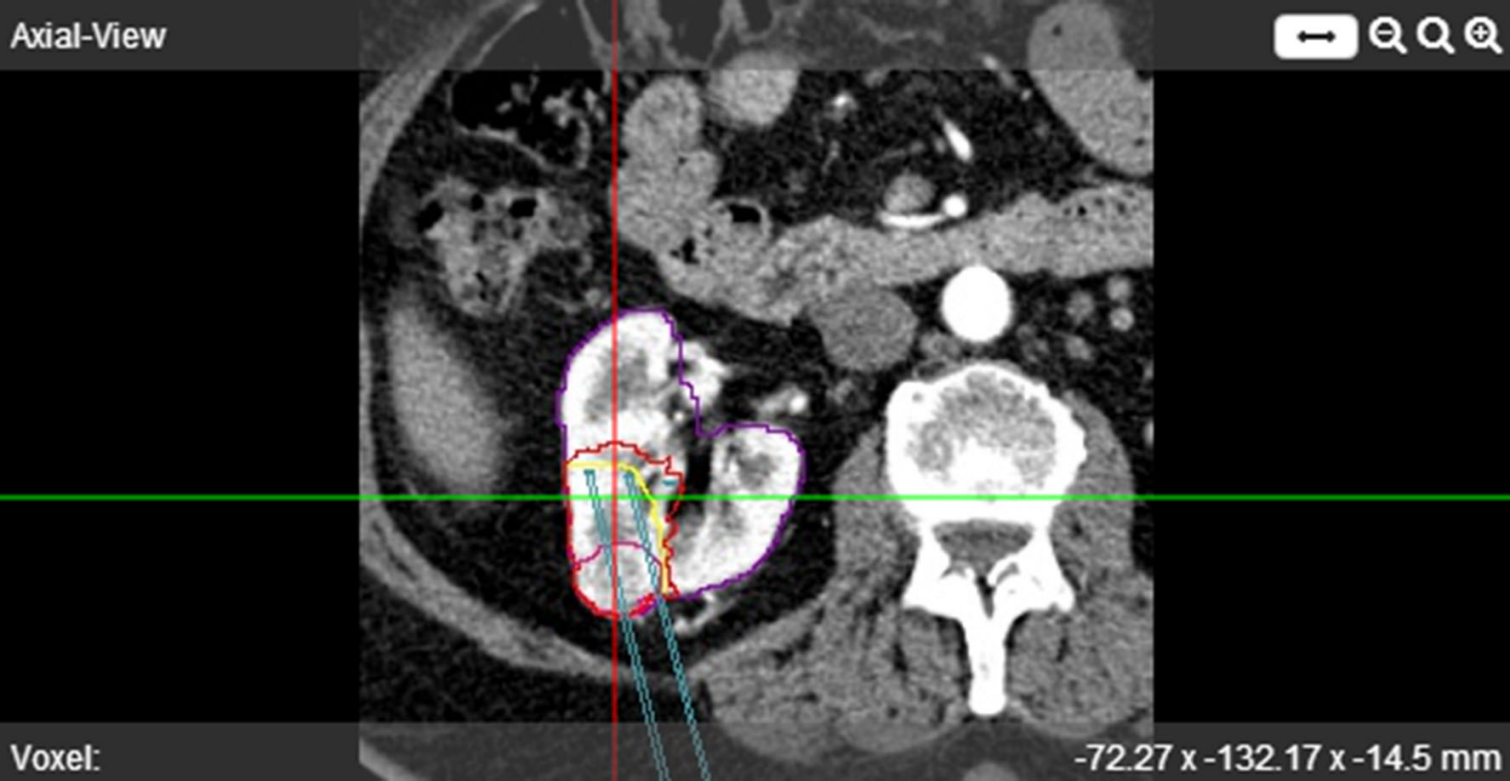
Contrast enhanced, pre-operative axial CT image in corticomedullary phase. Result of automated kidney (purple) and tumor (pink) segmentation are shown. The needles (blue) with original coordinates and simulation result (red) which are registered on to the pre-operative scan are shown as well. The real ablation zone segmented from the 1-month FU scan is shown in yellow (next steps). The simulated ablation zone is overestimated in this case

For cryoablation currently only the MRI Seednet® generator (Galil Medical) is available and validated in the environment. The needles IceSeed® and IceRod® can be used for the simulation. The default protocol is set to two cycles, each cycle contains 10 min of freezing, 2 min of passive thawing, and 1 min of active thawing. The freezing power can be adjusted per needle as a percentage of the maximum freezing power. The computer simulation takes approximately 10–15 min depending on the number and configuration of needles used. The generated outcome after the simulation shows quantified coverage of the segmented tumor (Fig. 5).

### Workflow for Validation of the Simulation Model

**Fig. 6:**
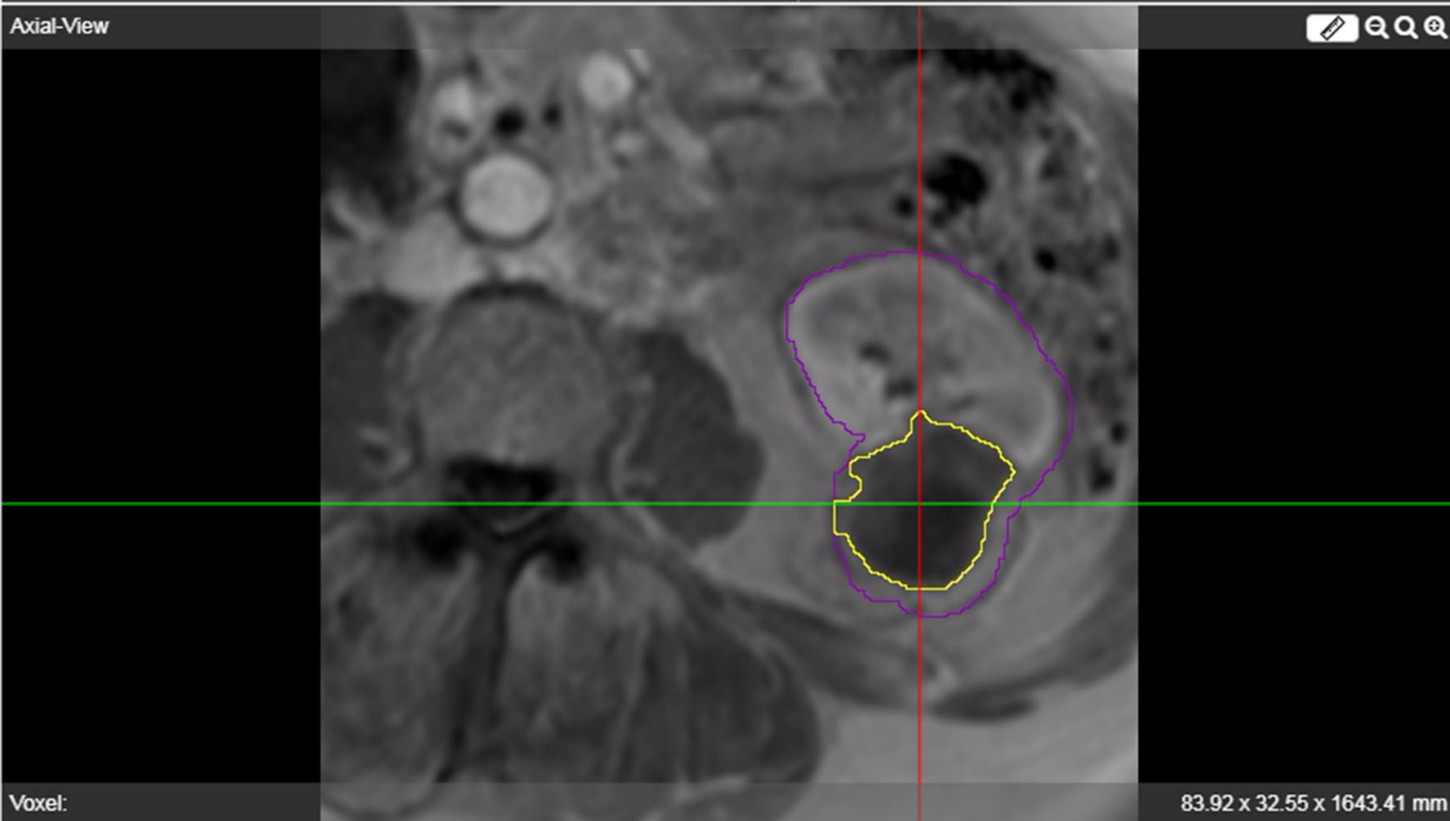
Example of segmented kidney (purple) and ablated ablation zone (yellow) on 1-month follow-up MRI scan

Validation requires intra-procedural images that allow accurate needle localization and follow-up images clearly demarking the actually ablated zone which is used as the reference standard. The validation tool within the environment is used to quantify the performance of the simulation tool. Step one is the registration of the intra-operative and follow-up scans to the pre-operative scan including segmentation of the kidney and tumor. Subsequently, real needle coordinates were obtained from the intra-operative scan and used to position the virtual needles after which a simulation is performed. A 1-month follow-up scan is used to segment the real ablation zone (Fig. 6). In this study, imaging parameters for follow-up imaging were standardized and thus consistent. The simulated ablation zone is compared to the segmented real ablation zone using a validation tool integrated into the environment as described below.

### Patient Selection

Informed consent was waived by the Institutional Review Board. All patients who underwent renal cryoablation between May 2014 and July 2017 (duration of the project) and did not opt-out for the use of anonymous data were included. No further exclusion criteria regarding the studied population were used. For validation of the simulation model, data from 19 procedures in 18 consecutive patients treated with percutaneous cryoablation for a renal tumor ≤ 4.5 cm were used. Procedural details are published elsewhere [16]. Demographics are described in Table 1.

**Table 1:** Demographics, tumor characteristics, and treatment specifications (19 tumors in 18 patients)

	*N* (%)/median (range)/ ± SD
Age (years)	72 (48–84)
Female	7 (35%)
Maximum tumor diameter (mm)	27 (12–44)
Left renal tumor	12 (60%)
Histology	
Clear cell	10 (52%)
Papillary	3 (16%)
Oncocytoma	3 (16%)
Inconclusive	3 (16%)
Needles used	3 (2–4)
Tumor location	
Anterior	7 (37%)
Posterior	12 (63%)
Growth pattern	
> 50% exophytic	14 (74%)
< 50% exophytic	5 (26%)

### Evaluation Parameters

The validation metrics for the surface and volumetric overlap between the real ablation zone (*S*) and the simulated ablation zone (Σ) were determined. For surface comparison, absolute average error (AEE) was used. This calculates the distance between the (topological) surfaces of *S* and Σ. An AAE of 3 mm was considered to represent a good match. The main validation metric of volumetric overlap was determined by the Dice Similarity Coefficient (DSC) calculated as (2*|*S* ∩ Σ|)/(|*S*| +|Σ|). This is the ratio between twice the overlapping volume of *S* and ∑, divided by the sum of the volume of both *S* and ∑ [17]. DSC has a restricted range of [0, 1], with a DSC = 0 indicating no overlap; and DSC = 1 indicating complete overlap of the simulated ablation zone over the real ablation zone. A ratio > 0.7 is generally considered a good alignment between the ablation zones [18].

Since DSC is a symmetric metric, it cannot be used to quantify either over- or underestimation of *S*. The following metrics were therefore computed [19]:

- Target overlap (TO) calculated as (|*S* ∩ Σ|)/(|*S*|). This is the ratio between the overlapping volume of *S* and Σ to the volume of *S*. A low ratio means more underestimation of *S* by the simulation model.
- Positive predictive value (PPV) calculated as (|*S* ∩ Σ|)/(|Σ|). This is the ratio between the overlapping volume of *S* and Σ to the volume of Σ. A low ratio means more overestimation of *S* by the simulation model.

Similar to the DSC, the TO and PPV have a restricted range from 0, indicating no overlap, to 1, indicating perfect overlap. The validation metrics were stratified based on an ordinal scale ranging between poor (value < 0.2) and excellent (value ≥ 0.8) (Table 2).

**Table 2:** Quantitative volumetric parameter ratings

Score	Ratio	DSC *n* (%)	TO *n* (%)	PPV *n* (%)
Excellent	1 > value ≥ 0.8	2 (10%)	16 (85%)	3 (16%)
Good	0.8 > value ≥ 0.7	7 (37%)	1 (5%)	1 (5%)
Adequate	0.7 > value ≥ 0.6	2 (11%)	1 (5%)	2 (11%)
Inadequate	0.6 > value ≥ 0.5	3 (16%)	1 (5%)	4 (21%)
Poor	Value < 0.5	5 (26%)	0	9 (47%)

*DSC* DICE similarity coefficient, *TO* target overlap, *PPV* positive predictive value

### Statistical Analysis

Descriptive statistics were performed using SPSS (version 22.0; IBM; Amonk; New York), i.e. medians and ranges or means and standard deviations were calculated.

## Results

In 18 patients, 19 MR-guided percutaneous cryoablations were performed. One patient was treated twice for the recurrent disease at different anatomical locations with an interval of 21 months. A median of 3 (range, 2–4) needles per procedure was used. All tumor characteristics and treatment specifications are listed in Table 1.

**Fig. 7:**
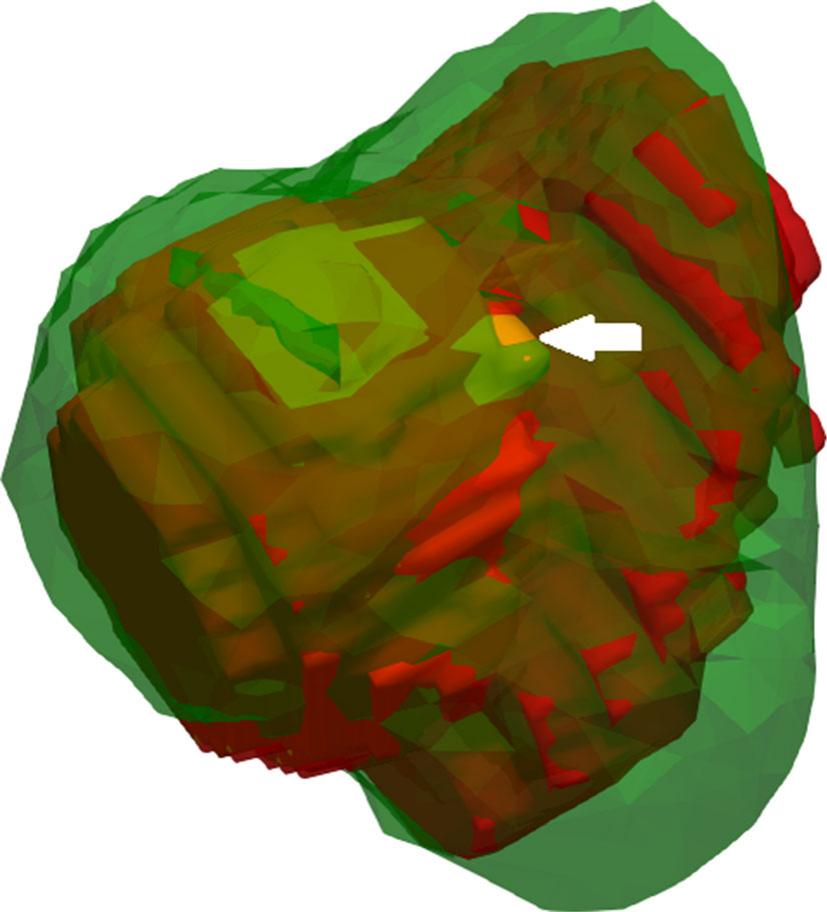
Example of validation case showing the simulated (Σ) ablation zone in green (volume 19.47 cm^3^) and segmented (*S*) ablation zone in red (volume 13.64 cm^3^). Darkgreen represent an overlap between Σ and *S* (green and red). A small part of the segmented tumor is visible in yellow (white arrow) and is not covered by both *S* and Σ. Average absolute error is 1.85 mm. DICE similarity coefficient and sensitivity were both scored as excellent with values of 0.8 and 0.95, respectively. Positive predictive value was scored as adequate with a value of 0.67

**Fig. 8:**
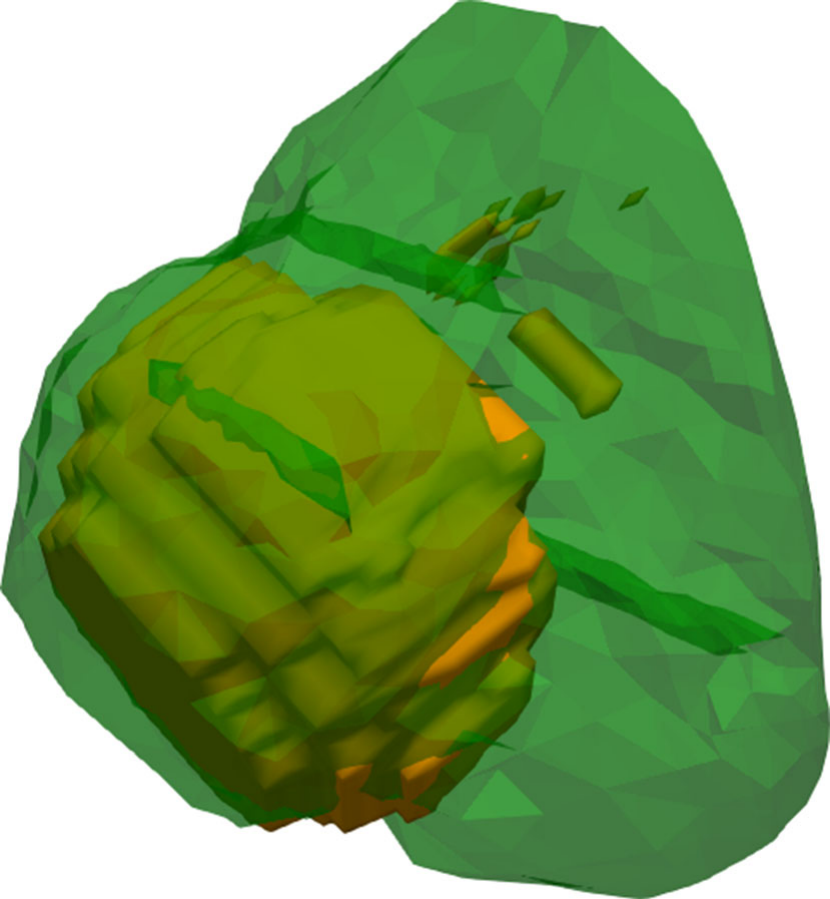
Only the simulated ablation zone (*S*; green) and tumor (yellow) are shown, as would be the case when using the environment for planning purposes

Mean volume of *S* (real ablation zone) and Σ (simulated ablation zone) was 14.8 cm^3^ (SD ± 9.9) and 26.6 cm^3^ (SD ± 15.4), respectively. The mean diameter of the sphere which circumscribes *S* was 4.6 cm (SD ± 1.1) and 5.6 cm (SD ± 1.3) for Σ. The AAE had a mean of 3.8 mm (SD ± 2.4). The mean value for DSC was 0.62 (SD ± 0.17). In 9 out of 19 cases (47%), DSC was scored as good or excellent (value ≥ 0.7) (Table 2; Figs. 7, 8).

Mean TO and PPV were 0.88 (SD ± 0.10) and 0.53 (SD ± 0.24), respectively. In 17 cases (89%), TO was scored as good or excellent. For PPV, only 4 cases (21%) were scored as such, 13 cases (68%) were scored poor or inadequate (value < 0.6) (Table 2).

**Fig. 9:**
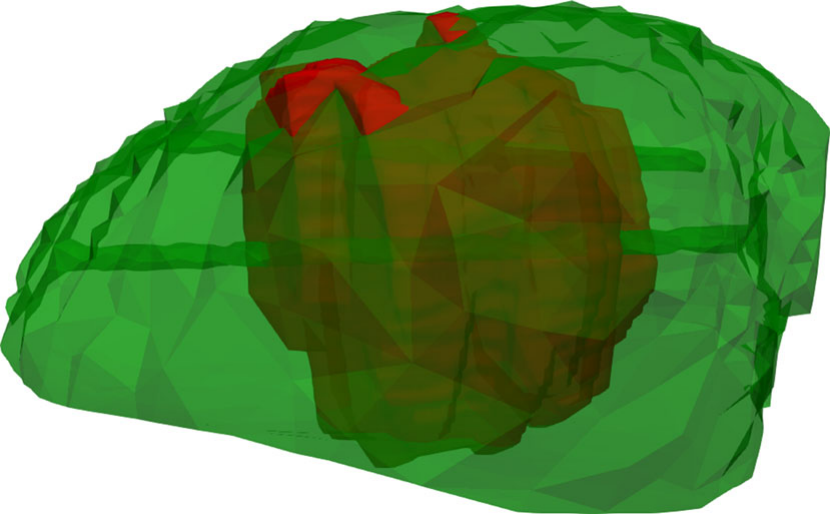
Example of case with overestimation. The simulated (Σ) ablation zone is shown in green (volume 43.49 cm^3^) and segmented (*S*) ablation zone in red (volume 10.95 cm^3^). Darkgreen represent an overlap between Σ and *S* (green and red). The needle tracts are visible in the ablation zones. Average absolute error is 6.72 mm. DICE similarity coefficient and positive predictive value were both scored as poor with values of 0.39 and 0.24, respectively. Sensitivity was scored as excellent with a value of 0.97

The relatively low values for PPV combined with high TO values indicate that the simulation is overestimating the real ablation zone in the majority of cases (Fig. 9).

## Discussion

In this study, a simulation model for renal cryoablation treatment planning was validated. Results from validation of the first 19 cases showed a tendency of overestimation of the real ablation zone by the simulation, leading to undertreatment when used in the clinical setting.

Based on this result, the simulation tool can be further refined. The inherent limitation to simulation models used for cryoablation is the development based on experimentally derived parameters, especially regarding bioheat transfer, which is generally recorded in an ex vivo setting [14]. Also, the additive effect of using multiple probes during the procedure is challenging to take into account based on the available literature. Because cryoablation modelling heavily depends on the application of accurate parameters, especially thermophysical properties, the use of experimentally derived parameters results in significant uncertainties in these models leading to errors when predicting the cryoablation treatment effect [13]. These errors become apparent during validation of the applied model in a setting as described in this study. Adjustment of the used parameters in the model is required for further refinement of the simulation tool. Due to a lack of available reliable tested parameters, subsequent testing on clinical cases as described in this study should reveal the sensitivity of the simulation model to the adjusted parameters. Optimizing the used physiological parameters should eventually lead to improved simulation results.

The complexity of predicting cryoablation effect underlines the importance of a simulation tool. The ablation effect is dependent on numerous physiological (e.g. blood perfusion, metabolic heat, thermophysical properties) and treatment protocol (e.g. duration freeze–thaw cycle) properties. The extent to which these properties affect the ablation effect is impossible to take into account purely by subjective evaluation of the treating interventionalist. Using a simulation tool taking these properties into account can facilitate treatment planning to ensure oncological safe ablation margins omitting unnecessary ablation of the healthy renal parenchyma.

Although the computational prediction of ice ball formation is complex, and mathematical models for prediction are continuously being improved, several planning tools for clinical use have been developed [9]. Boas et al. developed and validated a planning tool for multiple-probe cryoablation [20]. In this study, simulations with different numbers and configuration of needles were performed using the Pennes bioheat equation. The simulated ice-ball sizes, measured along 3 perpendicular axes, were validated using 26 gel experiments and 42 clinical kidney and liver cases. The surface deviation between the simulated and real ice-ball was used for validation, and showed an absolute average error of 4 mm in the clinical cases. This is comparable to our study (3.8 mm SD ± 2.8). In the treatment planning workflow presented by Boas et al., the desired ice-ball measurements are provided by the interventionalist after which the environment provides a number and configuration of needles resulting in an ice ball with the closest match. Compared to our planning tool, this model lacks a quantification of volumetric coverage between the predicted ablation zone and the tumor. Another limitation is that despite the implementation of a wide variety of needle configurations, the provided advice for needle configuration by this model may clinically be unfeasible. In the environment presented in this study, more flexibility is provided by enabling simulations with up to nine needles with an infinite number of configurations that can be evaluated for adequate tumor coverage.

Torricelli et al. described the development of an algorithm for planning the number and configuration of needles for cryoablation based on the spherical-shaped ice ball formation from one needle as provided by the manufacturer [21]. A stepwise approach was used starting by computing the initial number of ice balls necessary, followed by simulating the configuration of ice balls and optimizing this (i.e. changing needle position) to cover the complete tumor. Finally, an extra ice ball can be added to reach full tumor coverage. Limitation of this approach is the assumption that multiple probe usage only gives an additive effect on ice ball volume and the ablated ablation zone. However, the use of multiple needles has a synergistic effect, resulting in a larger effect than only additive [22, 23]. Moreover, the predicted ice ball size as provided by the manufacturer (tested in gels) tends to overestimate the ablation zone after treatment in vivo [24]. Also, this model has not been validated.

Treatment planning using a simulation model can have multiple purposes. The primary goal is to facilitate pre-procedural planning of image-guided percutaneous ablative therapies. This is done by enabling the interventionalist to virtual test the optimal needle type, number of needles used, and needle configuration in order to obtain complete tumor coverage. Second, the model can be used for training purposes by untrained interventionalists to become acquainted with the treatment effects. Third, it would be beneficial to have an environment enabling the comparison of several ablative treatment modalities, such as microwave ablation (MW), radiofrequency ablation (RFA), and irreversible electroporation (IRE) to choose the optimal treatment modality for individual treatments. Within the Go-Smart project, of which the development of the cryoablation planning tool was a part, also simulation tools for microwave (MWA)- and radiofrequency ablation (RFA) and irreversible electroporation (IRE) for several organs were developed simultaneously. However, preliminary testing during the development phase showed the most favorable results for cryoablation. Most challenging was the development of MWA due to the complexity of electromagnetic modelling, and IRE due to the challenging correct determination of an ablation zone [14]. Improvement of the simulation tools for the other treatment modalities and subsequent validation is awaited.

Artificial intelligence algorithms can directly test model sensitivity to adjustment of thermophysical parameters to evaluate what parameter adjustment optimizes the simulation results can be of great benefit during model validation. Also, algorithms for automated image registration and segmentation would be helpful to increase the speed and accuracy of the model, but moreover would facilitate rapid treatment effect evaluation in clinical use once the ablation is performed.

Some limitations were present that influenced our simulation results. An important limitation is heterogeneity between histological tumor types, e.g. perfusion characteristics, which were not adjusted for in the simulation model. Also the simulation interface suggests a fully rigid needle position. A wide variety of factors, such as breathing or slight needle repositioning between ablation cycles, can lead to needle movements. In a retrospective analysis, it is impossible to account for these movements. Furthermore, deviations in needle alignment between needle position and registered needle position may occur in a millimeter fashion due to artifacts on imaging, errors in needle identification, and registration inaccuracy. Although validation results were not optimal so far, sources of error were investigated and identified. Adjustment of the simulation model for these errors will benefit future simulation results.

## Conclusion

In this study, we validated a simulation model used for renal tumor cryoablation treatment planning within a web-based environment. Based on the first validation results of the simulation model, we conclude that refinement of the simulation model is needed to reduce overestimation of the ablation effect. Model parameters adjustment to improve simulation performance and evaluation of the adjustment effect are possible within the environment. Until more accurate results are obtained, the simulation model is not suitable for use in clinical practice.
